# Killing of *Serratia marcescens* biofilms with chloramphenicol

**DOI:** 10.1186/s12941-017-0192-2

**Published:** 2017-03-29

**Authors:** Christopher Ray, Anukul T. Shenoy, Carlos J. Orihuela, Norberto González-Juarbe

**Affiliations:** Department of Microbiology, The University of Alabama at Birmingham, Birmingham, AL USA

**Keywords:** *Serratia marcescens*, Biofilm, Antibiotics, Chloramphenicol

## Abstract

*Serratia marcescens* is a Gram-negative bacterium with proven resistance to multiple antibiotics and causative of catheter-associated infections. Bacterial colonization of catheters mainly involves the formation of biofilm. The objectives of this study were to explore the susceptibility of *S. marcescens* biofilms to high doses of common antibiotics and non-antimicrobial agents. Biofilms formed by a clinical isolate of *S. marcescens* were treated with ceftriaxone, kanamycin, gentamicin, and chloramphenicol at doses corresponding to 10, 100 and 1000 times their planktonic minimum inhibitory concentration. In addition, biofilms were also treated with chemical compounds such as polysorbate-80 and ursolic acid. *S. marcescens* demonstrated susceptibility to ceftriaxone, kanamycin, gentamicin, and chloramphenicol in its planktonic form, however, only chloramphenicol reduced both biofilm biomass and biofilm viability. Polysorbate-80 and ursolic acid had minimal to no effect on either planktonic and biofilm grown *S. marcescens*. Our results suggest that supratherapeutic doses of chloramphenicol can be used effectively against established *S. marcescens* biofilms.

**Electronic supplementary material:**

The online version of this article (doi:10.1186/s12941-017-0192-2) contains supplementary material, which is available to authorized users.

## Background

*Serratia marcescens* is a Gram-negative bacterium that causes infections in plants, insects, and animals, including humans [1]. Initially believed to be non-pathogenic, *S. marcescens* is currently known to cause ocular, pulmonary, urinary tract, and blood-stream infections [1, 2]. Recent reports implicate this pathogen in 2.5–7.7% of catheter-associated infections such as central line-associated bloodstream infections (CLABSI) and catheter-associated urinary tract infections [3–5]. In the United States alone, an estimated 80,000 catheter-associated bloodstream infections occur every year with costs up to $56,000 per episode [6]. Bacterial colonization of catheters confers a survival advantage and leads to the formation of cooperative bacterial communities known as “biofilms” [7]. Bacterial biofilms are complex surface-adhered communities of viable and dead bacteria, encased within an extracellular matrix composed of polysaccharide, protein and extracellular DNA [8]. In addition, bacteria adhere to host’s epithelial cells through formation of biofilm [9, 10]. The ability of *S. marcescens* to form biofilms contributes to its pathogenicity [1, 11]. Bacteria in biofilms also exhibit increased resistance to antimicrobial agents for reasons such as resistance to antibiotic penetration and metabolic changes such as slower growth rates [7, 12].

One current treatment used for salvaging biofilm-colonized catheters implicated in CLABSI is the application of antibiotic lock therapy (ALT). During ALT, supratherapeutic concentrations of antibiotic solutions are locked in the affected catheter for an extended amount of time [13, 14]. However, to our knowledge, the effect of ALT on *S. marcescens* biofilms has not yet been studied extensively in vivo nor in vitro. Another biofilm-control strategy centers on chemicals with no antimicrobial activity, which affects biofilm formation and adhesion by alteration of the bacterial microenvironment [12]. Recent reports have demonstrated the efficacy of polysorbate-80 (PS80) a nonionic surfactant, in reducing the biofilm mass of *Escherichia coli*, *Stenotrophomonas maltophilia*, *Listeria monocytogenes*, and *Pseudomonas fluorescens* [15–17]. PS80 is a solubilizing and emulsifying agent used in ointments, lotions, soaps as well as in medical preparations such as vitamin oils and anticancer agents [15]. In addition to PS80, ursolic acid (UA) is a plant-derived agent shown to reduce *Actinomyces viscosus*, *Streptococcus mutants*, *Vibrio harveyi*, and *Pseudomonas aeruginosa* biofilm mass [18, 19]. Herein, we determined the effectiveness of high-dose antibiotic, PS80 and UA therapies on in vitro biofilms formed by a clinical isolate of *S. marcescens*.

## Methods

### Planktonic bacterial growth

The tetracycline-resistant *S. marcescens* clinical isolate UT-383 was obtained from Dr. Jan Patterson (Division of Infectious Disease, Department of Medicine, The University of Texas Health Sciences Center at San Antonio) [2]. *S. marcescens* was grown on Luria–Bertani (LB) agar plates (LB-Lennox formulation) and incubated overnight at 37 °C. A single colony was transferred to LB broth and incubated overnight at 37 °C. The culture was then back diluted in fresh LB broth and incubated for 3 h at 37 °C to an optical density (OD_621_) of 0.5 nm. To establish the minimum inhibitory concentration (MIC) of ceftriaxone, kanamycin, gentamicin and chloramphenicol on *S. marcescens.* Planktonic grown bacteria were exposed to the latter antibiotics, plates were incubated overnight, and MIC was determined as the lowest concentration of antibiotics with no growth detected by spectrophotometry at OD_621_.

### Biofilm growth and quantification

Biofilm growth was initiated by inoculating 4 ml of LB broth with 1 × 10^5^ CFU of *S. marcescens* using a 6-well polystyrene plate (Corning Inc., Corning NY). Cultures were grown overnight at 37 °C. 3 ml of media were then gently aspirated and replaced with 1 ml of LB and 2 ml of Mueller–Hinton broth (MH) (control) or MH-based antibiotic solutions (ceftriaxone: 0.005, 0.05, 0.5 mg/ml; kanamycin: 0.625, 6.25, 62.5 mg/ml; gentamicin: 0.08, 0.80, 8.0 mg/ml; and chloramphenicol: 0.08, 0.80 mg/ml). Solutions of PS80 were prepared to yield final concentrations of 1, 0.1, and 0.01%, and UA to yield a final concentration of 0.03 mg/ml. Biofilms were then returned to the incubator overnight. The media on each well was then aspirated and biofilms stained with 1% crystal violet for 10 min, washed twice with 2 ml of 1× PBS, dried, and photographed. A representative image of each biofilm was taken with a Leica LMD6 inverted microscope with a DFC3000G-1.3-megapixel monochrome camera (Leica Biosystems, Buffalo Grove, IL). Bacterial biofilms were then solubilized with 2 ml of 95% ethanol, rocked for 30 min and the optical density at 540 nm of a 1:10 ethanol dilution was measured using an iMark Absorbance Microplate Reader (Bio-Rad Laboratories, Hercules, CA). Controls of each antibiotic were incubated sterile overnight, stained, solubilized, and read in the same manner to account for background staining from each concentration of the solution.

Absorbance values were calculated by subtracting the background stain from each well. Values were then normalized to 1 by dividing by the mean growth of the bacteria-only controls of each experiment (which were calculated by subtracting the mean of all media-only wells from each bacteria-only control). A total of three experiments were carried out with ceftriaxone, kanamycin, and gentamicin (5 total replicates), while four experiments were carried out with chloramphenicol, PS80, and UA (7 total replicates).

### Viability of biofilms treated with chloramphenicol or PS80

After 24 h of growth, biofilms were treated with chloramphenicol or PS80 and incubated for an extra 24 h at 37 °C. Biofilms were then washed gently with 2 ml of sterile PBS to dislodge remaining planktonic bacteria, and dried overnight at room temperature. Subsequently, 1 ml of sterile PBS was added to each well and biofilms removed by scraping. The supernatant was serially diluted in PBS, then 100 µl of each dilution was plated on individual blood agar petri dishes and incubated overnight to quantify the viable colony-forming units in the biofilm [2, 20].

### Antibiotics and other chemicals

Antibiotic solutions were made in Mueller–Hinton broth (MH). Ceftriaxone (Sigma-Aldrich, St. Louis MO), kanamycin (Sigma-Aldrich), gentamicin (Gibco, Grand Island NY), and chloramphenicol (Sigma-Aldrich). Polysorbate-80 (Fisher Scientific, Fair Lawn NJ) and ursolic acid (Sigma-Aldrich).

### Statistical analysis

Each set of experiments was then analyzed separately using Kruskal–Wallis ANOVA with multiple comparisons on GraphPad Prism version 7.0a (La Jolla CA).

## Results

### High concentrations of chloramphenicol reduce biofilm growth of *S. marcescens*

**Fig. 1:**
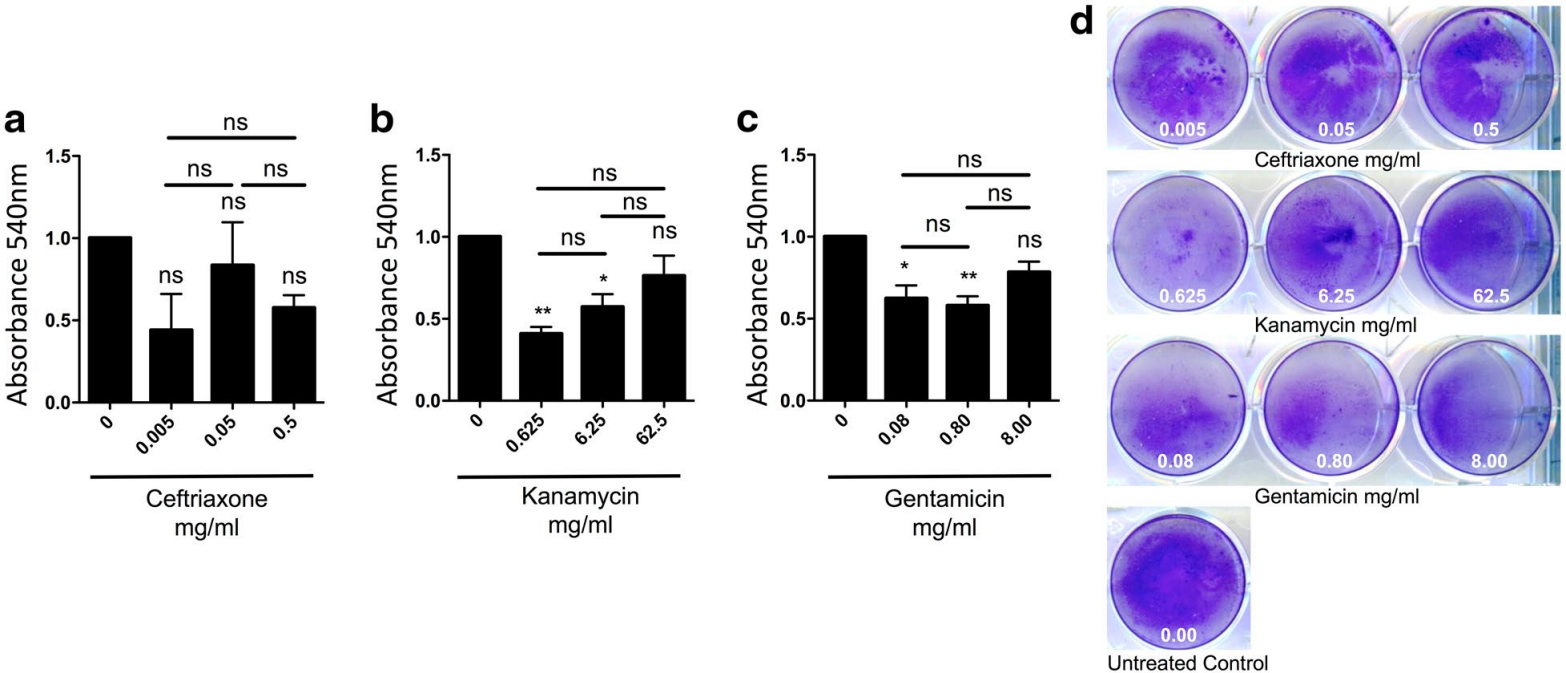
*S. marcescens* biofilms are partially resistant to ceftriaxone, gentamicin and kanamycin. **a**–**c** *S. marcescens* biofilms were treated with **a** ceftriaxone: 0.005, 0.05, 0.5 mg/ml; **b** kanamycin: 0.625, 6.25, 62.5 mg/ml; and **c** gentamicin: 0.08, 0.80, 8.0 mg/ml. Absorbance (540 nm) for antibiotic experiments (mean + SD). Values are normalized to the mean control of each experiment. **d** Representative pictures of in vitro *S. marcescens* biofilms treated with ceftriaxone, kanamycin or gentamicin at shown concentrations. For multi-group comparisons Dunn’s multiple-comparison post-test was used: *P < 0.05, **P ≤ 0.01, ***P ≤ 0.001

To test the susceptibility *S. marcescens* to multiple antibiotics, we co-cultured the latter in the presence of ceftriaxone, kanamycin, gentamicin, chloramphenicol, tetracycline, and erythromycin. Planktonic *S. marcescens* demonstrated susceptibility to all tested antibiotics except tetracycline and erythromycin at various doses (Additional file 1: Table S1). We then tested the ability of the antibiotics mentioned above to reduce *S. marcescens* biofilm biomass in vitro. *S. marcescens* biofilms were treated with ceftriaxone, kanamycin, gentamicin, and chloramphenicol at 10, 100, and 1000 times the planktonic minimum inhibitory concentration (MIC, see Additional file 2: Table S2). Biofilm biomass showed no change after treatment with 0.005, 0.05, 0.5 mg/ml of ceftriaxone (Fig. 1). At the lowest tested concentration of kanamycin (0.625 mg/ml) biofilm biomass was reduced. However, at 62.5 mg/ml of kanamycin, no change in biofilm formation was observed. Similar results also occurred when gentamicin was used (Fig. 1). These findings suggest a lack of dose-dependent effect in the reduction of *S. marcescens* biofilm biomass by kanamycin, gentamicin, and ceftriaxone; and raise the possibility that antibiotics promote biofilm formation.

**Fig. 2:**
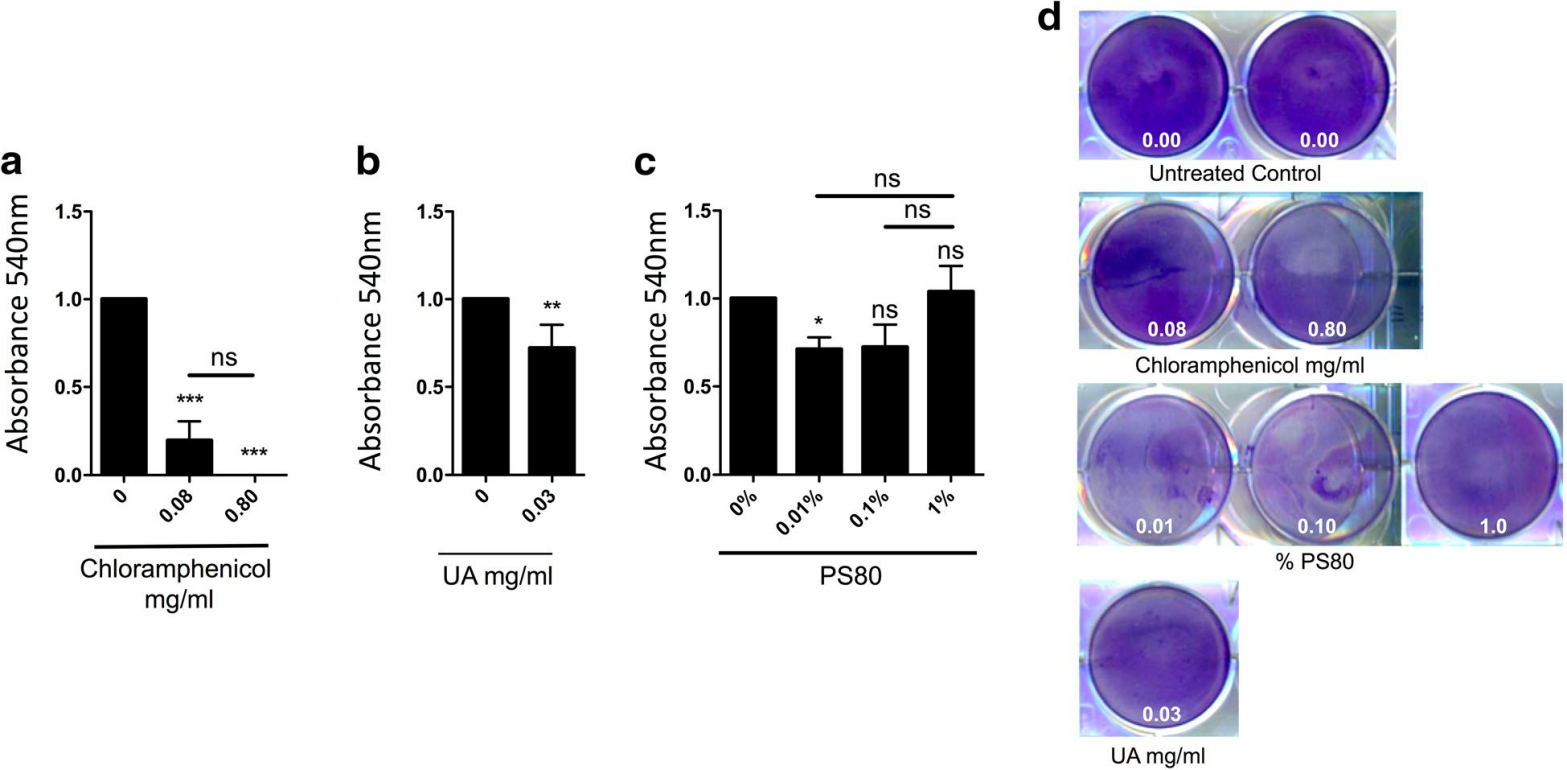
Chloramphenicol but polysorbate-80 or ursolic acid reduces *S. marcescens* biofilm biomass. **a**–**c** *S. marcescens* biofilms were treated with **a** chloramphenicol: 0.08, 0.80 mg/ml, **b** ursolic acid at 0.03 mg/ml or **c** 1, 0.1 0.01% polysorbate-80 (PS80). **d** Representative pictures of in vitro *S. marcescens* biofilms treated with chloramphenicol, polysorbate-80 (PS80) and ursolic acid (UA) at shown concentrations. Absorbance (540 nm) for chemical experiments (mean + SD), values are normalized to the mean control of each experiment. For multi-group comparisons Dunn’s multiple-comparison post-test was used: *P < 0.05, **P ≤ 0.01, ***P ≤ 0.001

Chloramphenicol is a last resort antibiotic used to treat infections such as tetracycline-resistant cholera [21] or brain abscesses caused by bacteria [22–24]. To determine if high doses of chloramphenicol could reduce biofilm biomass we co-cultured *S. marcescens* with 0.08 and 0.80 mg/ml of the latter antibiotic. Biofilm mass as determined by crystal violet staining showed a significant decrease at both 0.08 mg/ml (P = 0.0003) and 0.80 mg/ml (P < 0.0001) of chloramphenicol, with a greater reduction at 0.80 mg/ml indicating dose-dependence (Fig. 2). Finally, biofilms treated with either PS80 or UA had a minimal but not significant change in biomass (Fig. 2). Collectively, these results suggest that antibiotics commonly used for ALT are not sufficient to reduce biomass of biofilms formed by a clinical isolate of *S. marcescens* except for chloramphenicol.

### PS80 1% and high dose chloramphenicol lessen the viability of *S. marcescens* biofilms

**Fig. 3:**
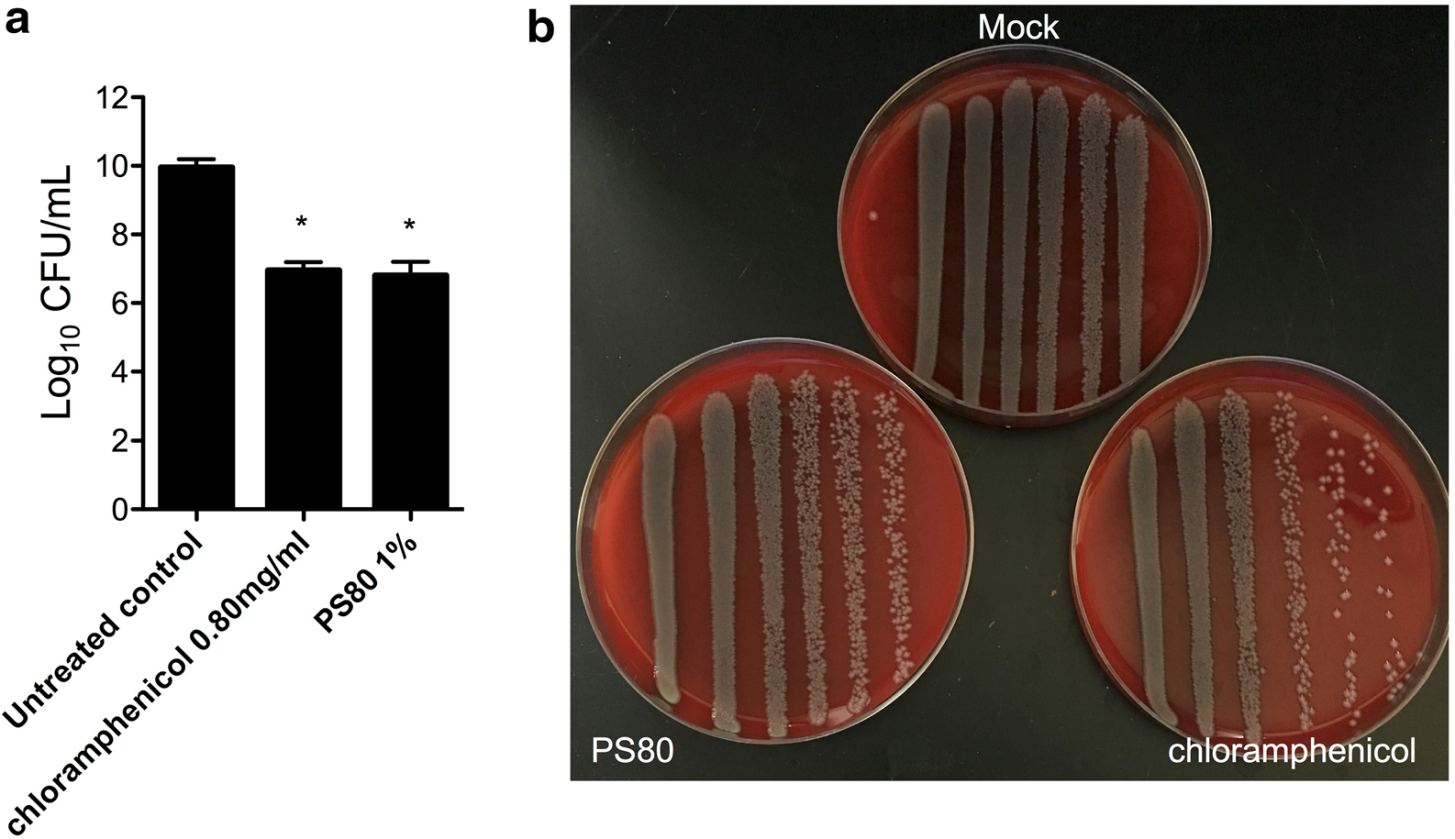
Polysorbate-80 and chloramphenicol reduce biofilm viability. **a** Log_10_ CFU of *S. marcescens* recovered after chloramphenicol (0.80 mg/ml) or polysorbate-80 (PS80, 1%) treatment of biofilms. **b** Representative images of *S. marcescens* recovered after chloramphenicol or polysorbate-80 treatment of biofilms. *Top*-untreated control; *Bottom left*-PS80; *Bottom right*-chloramphenicol. Chloramphenicol (0.80 mg/ml) and PS80: P < 0.05. For multi-group comparisons Dunn’s multiple-comparison post-test was used: *P < 0.05, **P ≤ 0.01, ***P ≤ 0.001

Quantification of viable bacteria from in vitro biofilms treated with both PS80 and chloramphenicol showed a significant reduction in the number of colony-forming units recovered after treatment (Fig. 3). These results suggest that PS80 and chloramphenicol could not only decrease *S. marcescens* biofilm biomass but also its viability.

## Discussion

The clinical guidelines from the Infectious Disease Society of America recommends the use of ALT for catheter salvage [25]. Our study suggests that ALT is a viable strategy against biofilms generated by a clinical isolate of *S. marcescens* depending on the selected antibiotics. The response to chloramphenicol and small doses of aminoglycosides offer two possible avenues to treat *S. marcescens* contaminated catheters and reduce infections associated with this organism.

A major limitation of our study is that only one strain of *S. marcescens* was tested against our panel of antibiotics. To date, 8 out of 14 species of *Serratia* are documented to be related to human infections [1]. *S. marcescens* is known to be endogenously resistant to antibiotics such as colistin and cephalothin [26]. In the hospital setting bacteria from the *Enterobacteriaceae* family have demonstrated to be extraordinary in their capability to acquire, transfer and express antimicrobial resistance [27]. For these reasons, another limitation of this report is that it might not represent all strains of *S. marcescens* as our clinical isolate might have gained other antimicrobial resistances in the healthcare setting not present in reference strains. Future studies are warranted to address these and other limitations. We are particularly interested in exploring the possibility that some antimicrobials might be promoting biofilm formation.

Chloramphenicol effectivity against bacterial biofilms is still not well defined [23]. A recent report found no anti-biofilm activity of chloramphenicol against *Staphylococcus aureus*, *E. coli*, and *Micrococcus luteus*. Importantly, the authors only used a concentration of only 0.005 mg/ml, a concentration well below the planktonic MIC for our bacterium and only slightly higher than the MICs for their three isolates (maximum 0.0031 mg/ml) [28]. In contrast, our study shows that high doses (0.08 and 0.80 mg/ml) of chloramphenicol reduce the mass of preformed *S. marcescens* biofilms, and the 0.80 mg/ml concentration decreases the viability of biofilms compared with untreated controls. Together these results carry important implications for its potential in ALT. Importantly, since chloramphenicol is barely soluble in water, small amounts of methanol were added to facilitate solubility (yielding a final concentration of <5% methanol in the 0.80 mg/ml solution, and <0.5% in the 0.08 mg/ml solution); however, it seems unlikely that these small amounts of methanol influenced biofilm mass.

The use of chloramphenicol in systemic therapy has been limited by its toxicity, including fatal occurrences of granulocytopenia, aplastic anemia, and thrombocytopenia. The limitations mentioned above are avoided in ALT since the removal of the solution from the catheter minimizes systemic exposure to antibiotics [28]. The broad spectrum of action, including against some multi-drug resistant bacteria, makes chloramphenicol an attractive candidate for treating CLABSI. Additionally, chloramphenicol is inexpensive and remains stable in the presence of heparin (which is commonly included in ALT to prevent catheter thrombosis). Further studies will be needed to examine the potential of chloramphenicol as a clinical treatment with other pathogens.

## Conclusions

In our study, biofilms formed by a clinical isolate of *S. marcescens* were only killed by the use of chloramphenicol at ten and one hundred times concentrations used to kill planktonic bacteria, non-other of the antibiotics tested had the same effect. These suggest that chloramphenicol might be utilized for ALT against not only *S. marcescens* but also other Gram-negative nosocomial pathogens.

## Additional files

**Additional file 1: Table S1.** Planktonic minimum inhibitory concentration for *S. marcescens* (mg/mL).

**Additional file 2: Table S2.** Chemical Concentrations Tested.

## Abbreviations

MIC: minimum inhibitory concentration
LB: Luria–Bertani media
PS80: polysorbate-80
UA: ursolic acid
CLABSI: central line-associated bloodstream infection
ALT: antibiotic lock therapy
OD: optical density
MH: Mueller–Hinton broth
