# Supplementary material for: Killing of Serratia marcescens biofilms with chloramphenicol
Source: Ann Clin Microbiol Antimicrob. 2017 Mar 29;16:19. doi: 10.1186/s12941-017-0192-2 (PMC5370475; doi:10.1186/s12941-017-0192-2)
Supplement: Supplementary file 1 — Additional file 1: Table S1. Planktonic minimum inhibitory concentration for S. marcescens (mg/mL). [file 12941_2017_192_MOESM1_ESM.pdf]

Table 1: Planktonic minimum inhibitory concentration for *S. marcescens* (mg/ml)

	Ceftriaxone	Kanamycin	Gentamicin	Chloramphenicol
S. marcescens	**0.0005**	**0.0625**	**0.008**	**0.008**
