# Supplementary material for: Killing of Serratia marcescens biofilms with chloramphenicol
Source: Ann Clin Microbiol Antimicrob. 2017 Mar 29;16:19. doi: 10.1186/s12941-017-0192-2 (PMC5370475; doi:10.1186/s12941-017-0192-2)
Supplement: Supplementary file 2 — Additional file 2: Table S2. Chemical Concentrations Tested. [file 12941_2017_192_MOESM2_ESM.pdf]

Table 2: Chemical Concentrations Tested

MIC	Ceftriaxone	Kanamycin	Gentamicin	Chloramphenicol	PS80	UA
**10**	0.005mg/ml	0.625mg/ml	0.08mg/ml	0.08mg/ml	0.01mg/ml	0.03mg/ml
**100**	0.05mg/ml	6.25mg/ml	0.80mg/ml	0.80mg/ml	0.10mg/ml	
**1000**	0.5mg/ml	62.5mg/ml	8.0mg/ml		1.00mg/ml	
